# *S*upported exercise *T*r*A*ining for *M*en w*I*th prostate ca*N*cer on *A*ndrogen deprivation therapy (STAMINA): study protocol for a randomised controlled trial of the clinical and cost-effectiveness of the STAMINA lifestyle intervention compared with optimised usual care, including internal pilot and parallel process evaluation

**DOI:** 10.1186/s13063-024-07989-y

**Published:** 2024-04-12

**Authors:** Emma McNaught, Sophie Reale, Liam Bourke, Janet E. Brown, Michelle Collinson, Florence Day, Jenny Hewison, Amanda J. Farrin, Saïd Ibeggazene, Aidan Q. Innes, Ellen Mason, David Meads, Alison Scope, Chris Taylor, Steph JC. Taylor, Rebecca R. Turner, Derek J. Rosario, on behalf of the STAMINA co-investigators

**Affiliations:** 1 Clinical Trials Research Unit, Leeds Institute of Clinical Trials Research, University of Leeds, Leeds, LS2 9JT UK; 2 Department of Allied Health Professions, Sheffield Hallam University, Sheffield, S10 2BP UK; 3 Division of Clinical Medicine, University of Sheffield, Sheffield, S10 2RX UK; 4 Division of Health Services Research, Leeds Institute of Health Sciences, University of Leeds, Leeds, LS2 9JT UK; 5 Nuffield Health, 2 Ashley Avenue, Epsom, Surrey KT18 5AL UK; 6 Academic Unit of Health Economics, Leeds Institute of Health Sciences, University of Leeds, Leeds, LS2 9JT UK; 7 Wolfson Institute of Population Health, Queen Mary University of London, Yvonne Carter Building, 58 Turner Street, London, E1 2AB UK; 8 Division of Psychology and Mental Health in the School of Health Sciences, The University of Manchester, Manchester, M13 9PL UK; 9 Department of Urology, Sheffield Teaching Hospitals NHS Foundation Trust, Royal Hallamshire Hospital, Glossop Road, Sheffield, S10 2JF UK

**Keywords:** Prostate cancer, Supervised exercise, Lifestyle intervention, Behaviour change, Complex intervention, Healthcare professionals, Exercise professionals, Randomised controlled trial, Process evaluation, Health economics

## Abstract

**Background:**

UK national clinical guidance recommends that men with prostate cancer on androgen deprivation therapy are offered twice weekly supervised aerobic and resistance exercise to address iatrogenic harm caused by treatment. Very few NHS trusts have established adequate provision of such services. Furthermore, interventions fail to demonstrate sustained behaviour change. The STAMINA lifestyle intervention offers a system-level change to clinical care delivery addressing barriers to long-term behaviour change and implementation of new prostate cancer care pathways. This trial aims to establish whether STAMINA is clinically and cost-effective in improving cancer-specific quality of life and/or reducing fatigue compared to optimised usual care. The process evaluation aims to inform the interpretation of results and, if the intervention is shown to benefit patients, to inform the implementation of the intervention into the NHS.

**Methods:**

Men with prostate cancer on androgen deprivation therapy (*n* = 697) will be identified from a minimum of 12 UK NHS trusts to participate in a multi-centre, two-arm, individually randomised controlled trial. Consenting men will have a ‘safety to exercise’ check and be randomly allocated (5:4) to the STAMINA lifestyle intervention (*n* = 384) or optimised usual care (*n* = 313). Outcomes will be collected at baseline, 3-, 6- and 12-month post-randomisation. The two primary outcomes are cancer-specific quality of life and fatigue. The parallel process evaluation will follow a mixed-methods approach to explore recruitment and aspects of the intervention including, reach, fidelity, acceptability, and implementation. An economic evaluation will estimate the cost-effectiveness of the STAMINA lifestyle intervention versus optimised usual care and a discrete choice experiment will explore patient preferences.

**Discussion:**

The STAMINA lifestyle intervention has the potential to improve quality of life and reduce fatigue in men on androgen deprivation therapy for prostate cancer. Embedding supervised exercise into prostate cancer care may also support long-term positive behaviour change and reduce adverse events caused by treatment. Findings will inform future clinical care and could provide a blueprint for the integration of supervised exercise and behavioural support into other cancer and/or clinical services.

**Trial registration:**

ISRCTN 46385239, registered on 30/07/2020. Cancer Research UK 17002, retrospectively registered on 24/08/2022.

**Supplementary Information:**

The online version contains supplementary material available at 10.1186/s13063-024-07989-y.

## Administrative information

**Table:** 

Title {1}	*S*upported exercise *T*r*A*ining for *M*en w*I*th prostate ca*N*cer on *A*ndrogen deprivation therapy – (STAMINA): study protocol for a randomised controlled trial of the clinical and cost-effectiveness of the STAMINA lifestyle intervention compared with optimised usual care, including internal pilot and parallel process evaluation
Trial registration {2a and 2b}	ISRCTN 46385239 (30/07/2020), Cancer Research UK 17002 (24/08/2022 – retrospectively registered)
Protocol version {3}	Protocol v10.0 05/07/2023
Funding {4}	NIHR Programme Grants for Applied Research (PGfAR) RP-PG-1016–20007
Author details {5a}	Emma McNaught^1^ (E.McNaught@leeds.ac.uk), Sophie Reale^2^ (S.Reale@shu.ac.uk), Liam Bourke^2^ (L.Bourke@shu.ac.uk), Janet E Brown^3^ (j.e.brown@sheffield.ac.uk), Michelle Collinson^1^ (M.Collinson@leeds.ac.uk), Florence Day^1^ (F.Day1@leeds.ac.uk), Jenny Hewison^4^ (j.hewison@leeds.ac.uk), Amanda J. Farrin^1^ (A.J.Farrin@leeds.ac.uk), Saïd Ibeggazene^2^ (S.Ibeggazene@shu.ac.uk), Aidan Q Innes^5^ (Aidan.Innes@nuffieldhealth.com), Ellen Mason^1^ (e-mason@live.com), David Meads^6^ (D.Meads@leeds.ac.uk), Alison Scope^2^ (Alison.Scope@shu.ac.uk), Chris Taylor^1^ (C.M.Taylor@leeds.ac.uk), Steph JC Taylor^7^ (S.J.C.Taylor@qmul.ac.uk), Rebecca R Turner^8^(Rebecca.Turner@manchester.ac.uk), Derek J Rosario^9^ (D.J.Rosario@Sheffield.ac.uk) ^1^Clinical Trials Research Unit, Leeds Institute of Clinical Trials Research, University of Leeds, Leeds, LS2 9JT ^2^Dept. of Allied Health Professions, Sheffield Hallam University, Sheffield, S10 2BP ^3^Division of Clinical Medicine, University of Sheffield, Sheffield, S10 2RX ^4^Division of Health Services Research, Leeds Institute of Health Sciences, University of Leeds, Leeds, LS2 9JT ^5^Nuffield Health, 2 Ashley Avenue, Epsom, Surrey, KT18 5AL ^6^Academic Unit of Health Economics, Leeds Institute of Health Sciences, University of Leeds, Leeds, LS2 9JT ^7^Wolfson institute of Population Health, Queen Mary University of London, Yvonne Carter Building, 58 Turner Street, London, E1 2AB ^8^Division of Psychology and Mental Health in the School of Health Sciences, The University of Manchester, Manchester, M13 9PL ^9^Sheffield Teaching Hospitals NHS Foundation Trust, Department of Urology, Royal Hallamshire Hospital, Glossop Road, Sheffield, S10 2JF
Name and contact information for the trial sponsor {5b}	Sheffield Teaching Hospitals NHS Foundation Trust: Clinical Research Office 0114 226 5943
Role of sponsor {5c}	The Sponsor had no direct input into the design or conduct of the trial and accepts no responsibility for the accuracy of content reproduced into documentation developed by collaborating or third-party organisations The funder had no role in trial design

## Introduction

### Background and rationale {6a}

Prostate cancer is common and responsible for a quarter of new male cancer diagnoses in the UK [1], with around 50% being locally advanced or metastatic at presentation. The prevalence of prostate cancer is likely to continue rising as a result of an ageing population, increased screening and advancements in treatment. However, for many men living with and beyond prostate cancer, persistent adverse effects from treatment are common, often debilitating and can be experienced lifelong [2].

Androgen deprivation therapy (ADT) remains the mainstay of treatment in prostate cancer as sole treatment in men unfit for any other treatment, combined with radiotherapy for locally advanced disease [3] and in conjunction with taxane-based chemotherapy or novel androgenic signalling inhibitors (e.g. abiraterone, enzalutamide, apalutamide and daralutamide or, in fitter patients, an androgenic signalling inhibitor in combination with taxane-based chemotherapy) for metastatic disease [4]. ADT is effective for prostate cancer and men can remain on ADT for up to two decades [5, 6]. However, ADT is associated with significant adverse events including hot flushes, fatigue, bone fracture risk, diabetes, cognitive dysfunction, depression, and sexual dysfunction [7, 8]. For example, 12 weeks of ADT have been shown to increase body fat by 5% and reduce lean body mass, predisposing men towards sarcopenia [9]. Furthermore, abdominal obesity, hyperglycaemia, and an increased risk of cardiovascular disease have been observed in men receiving ADT [10–13]. Subsequently, the impact of ADT adverse events on quality of life (QoL) [14] and healthcare/treatment costs [15] is increasingly recognised.

To date, exercise training is the only evidence-based intervention to demonstrate clinically relevant beneficial effects on fatigue and disease-specific QoL for men receiving ADT for prostate cancer [16]. Exercise is also associated with improvements in cardiovascular health and potential benefits for mental health for men on ADT [17, 18]. Consequently, UK (the National Institute for Health and Care Excellence (NICE) NG 131 1.4.19 [19]) and international (European Association of Urology; EAU [20]) guidelines recommend 12 weeks of combined resistance and aerobic exercise as standard treatment for people on ADT. However, the UK National Health Service (NHS) provision of such treatment is almost non-existent [21]. Furthermore, established short-term benefits of supervised exercise dissipate without ongoing support demonstrating the significant challenges associated with maintaining behaviour change [22].

The STAMINA lifestyle intervention (SLI) is a behaviourally informed supervised exercise programme with dietary support developed using intervention development methodology [23] and underpinned by evidence from an earlier programme development grant [21]. Healthcare professionals (HCPs) will be trained to endorse and refer men to SLI and provide ongoing behavioural support whilst undergoing ADT [24]. Supervised exercise will be delivered by trained exercise professionals (known as Clinical Exercise Specialists (CESs)) from Nuffield Health (NH); a UK healthcare charity that invests heavily in staff training and routinely delivers exercise and behavioural support to various clinical populations.

The SLI has demonstrated good feasibility, acceptability, fidelity, and safety when embedded into routine clinical practice with delivery partner NH during a feasibility study [25]. The STAMINA model could offer an improved standard of care for men with prostate cancer and provide a blueprint for the integration of supervised exercise and behavioural support into other cancer or clinical services. However, the long-term clinical and cost-effectiveness of the intervention is currently unknown.

### Objectives {7}

#### Primary objective

To examine the effect of SLI on cancer-specific QoL measured using the Functional Assessment of Cancer Therapy – Prostate (FACT-P [26]) or on cancer-specific fatigue measured using the Functional Assessment of Chronic Illness Therapy – Fatigue (FACIT-F [27]), both at 12-month post-randomisation.

#### Secondary objectives

- To establish whether the intervention promotes physical, social, emotional, and functional wellbeing at 3-, 6- and 12-month post-randomisation using FACT-P [26] subdomains.
- To establish whether the intervention reduces cancer-specific fatigue at 3- and 6-month post-randomisation using FACIT-F [27].
- To establish whether the intervention increases leisure time physical activity, measured using the Godin [28] questionnaire at 3-, 6-, and 12-month post-randomisation.
- To establish whether the intervention reduces fear of cancer recurrence measured using Fear of Cancer Recurrence (FCR4 and FCR7) [29] at 3-, 6- and 12-month post-randomisation.
- To establish whether the intervention improves functional capacity and body composition, measured using blood pressure, chair sit-to-stand, waist and hip circumference and weight at 3-, 6-, and 12-month post-randomisation.
- To establish related and unexpected serious adverse event (RUSAE) rates, and severity.
- To establish whether the SLI reduces adverse effects, commonly associated with ADT measured using a trial-specific ADT symptom index at 3-, 6- and 12-month post-randomisation.
- To establish participants’ perceptions of capabilities, opportunities, and motivations to perform a target behaviour using the Capability, Opportunity, Motivation-Behaviour (COM-B) [30] Questionnaire.
- To establish whether the intervention is cost-effective, assessed using incremental cost-effectiveness ratios (ICERs). The outcome of interest is cost per incremental quality-adjusted life year (QALY) at 12-month post-randomisation with QALYs being derived from the EuroQol 5 Domains 5 Levels (EQ-5D-5L) [31].
- To establish patient preferences and to predict demand for alternative extended exercise programme features using a discrete choice experiment (DCE) [32].
- To establish moderator and mediator variables which influence engagement with and benefit from treatment.

#### Internal pilot objectives

To assess rates of recruitment, 3-month follow-up, and adherence to the intervention, after 12 months of participant recruitment, against pre-defined progression criteria.

#### Process evaluation objectives

- To understand trial recruitment performance.
- To describe intervention reach, dose delivered, and dose received.
- To describe the fidelity of intervention delivery.
- To understand how the intervention was experienced and understood by patients using semi-structured interviews (underpinned by consideration of the Theoretical Framework of Acceptability).
- To explore the organisational implications of embedding and sustaining the intervention in preparation for wider NHS roll-out (underpinned by consideration of Normalization Process Theory).

### Trial design {8}

The STAMINA trial is a multi-centre, two-arm, individually randomised controlled trial (RCT) with a two-level partially nested design, an internal pilot with clear progression criteria, a cost-effectiveness analysis and a parallel mixed-methods process evaluation. Six hundred and ninety-seven participants will be randomly allocated with a 5:4 ratio to either SLI or optimised usual care (OUC).

## Methods: participants, interventions and outcomes

### Study setting {9}

Recruitment will take place across England at a minimum of 12 NHS Trusts (listed in the ‘Acknowledgements’ section). Potential trial participants will be approached to take part by secondary care clinical or research teams, and those willing and potentially eligible will be referred onto the Sheffield Hallam University research team for consent and baseline data collection. The SLI will be delivered in NH fitness and wellbeing centres (listed in the ‘Acknowledgements’ section) by CESs. Participants in both trial arms will receive OUC provided by STAMINA-trained secondary care NHS HCPs.

### Eligibility criteria {10}

#### Trial sites

NHS sites that previously expressed an interest in participating in the STAMINA programme as part of earlier work will be approached by the trial team and assessed for eligibility. A feasibility questionnaire will determine if sites have services appropriate to support recruitment, delivery of OUC, and support for trial-related research activity (e.g. data collection). Eligible sites include secondary or tertiary care hospitals with a urology diagnostic centre or a cancer treatment centre where men on ADT have their allocated consultant, that:

- Have at least one NH fitness and wellbeing centre located within reasonable travelling distance in the locality
- Agree to keep an ADT log to facilitate audit of the men’s treatment
- Identify the care team involved in delivering care to men with prostate cancer and make them available for training, and
- Agree to usual care for men on ADT eligible to exercise to become the STAMINA OUC arm.

Sites already delivering an embedded exercise intervention that is considered superior to OUC will be excluded.

#### Participants

Patients meeting all the following criteria (and none of the exclusion criteria) prior to randomisation will be eligible to take part:

##### Inclusion criteria

- Men with prostate cancer on ADT or due to start ADT within the next 12 weeks.^1^
- Willing to provide informed consent.

##### Exclusion criteria

- Absolute contraindication to exercise as defined by clinical guidance, e.g. ACPICR standards [33];
- Uncontrolled hypertension;
- Uncontrolled diabetes mellitus;
- Recent myocardial infarction (within the past 6 months);
- Unable to provide informed consent (e.g. lack of capacity);
- Unstable bony metastases unresponsive to treatment;
- Unable to complete study assessments;
- Participation in other lifestyle intervention trials for prostate cancer;
- Estimated life expectancy of less than 12 months for reasons unrelated to prostate cancer diagnosis; and
- Involvement in previous STAMINA work packages or patient and public involvement (PPI) panel.

Participation in another study will not necessarily exclude a patient from participation. The feasibility of co-enrolment will be reviewed, considering methodological impact and participant burden.

Documented reasons for ineligibility or declining participation will be monitored by the Leeds Clinical Trials Research Unit (CTRU) as part of a regular review of recruitment progress.

### Who will take informed consent? {26a}

Clinical staff at site will be required to complete an ADT log for men with prostate cancer receiving ADT within their diagnostic unit. HCPs will gain verbal consent from initially eligible patients for referral to the research team at Sheffield Hallam University (SHU) for a ‘safety to exercise’ check. Any uncertainties around suitability for exercise will be checked against participant’s medical records. The potential participant will be given written information about the trial, and the opportunity to ask any questions or raise any queries about participation. A trained member of the research team at SHU will obtain participant consent by telephone for trial participation and data collection. If the potential participant chooses to consent, the patient will be registered onto the trial. Following confirmation of eligibility and completion of baseline assessments the registered participant will be randomised to their treatment allocation.

#### Withdrawal of consent

Withdrawal from, or non-attendance for, the intervention are not classed as withdrawal from the trial, and all follow-up will continue as planned, unless a participant specifically expresses a wish to withdraw from trial processes. Clarification will be sought on whether withdrawal is from participation in the intervention, questionnaire completion or ongoing access to health records and data processing.

The right of a participant to refuse participation without giving reasons will be accepted. The participant will remain free to withdraw at any time from the trial without giving reasons and without prejudicing their further treatment. If participants of the proposed trial withdraw consent from further participation, their data collected up to that point will be included in the final trial analysis. This will be made clear to the participants at the time of consent and when they withdraw from the trial.

### Additional consent provisions for collection and use of participant data and biological specimens {26b}

Participant consent for information collected as part of the STAMINA trial to be shared for use in future research is optional in the original consent. Participant consent to wear a heart rate monitor and upload their anonymised data onto Garmin is optional in the original consent for SLI participants.

## Interventions

### Explanation for the choice of comparators {6b}

Optimised usual care was developed based on a) findings from the programme development grant and early work packages [21], exploring variations in NHS prostate cancer care pathways and exercise provision for men on ADT and b) from the SHU research teams’ experience of providing written guidance for control groups in cancer and lifestyle research [34].

### Intervention description {11a}

#### Optimised usual care (OUC)

All participants will receive OUC (including SLI participants). OUC includes endorsement of the NICE guidelines around the benefits of exercise for men on ADT and behavioural support (where appropriate) from key workers during routine clinic appointments. Consenting participants will then undergo a ‘safety to exercise’ check administered over the phone by researchers at Sheffield Hallam University, with support from clinical teams, principal investigators, and the chief investigator where clinical queries arise. Participants will be sent an information pack through the post including a booklet about the NICE guidelines for people on ADT and local and nationally available resources related to lifestyle (e.g. information materials from Prostate Cancer UK (PCUK) [35], Macmillan [36], Cancer Research UK (CRUK) [37]).

#### The healthcare professional intervention

NHS prostate cancer clinical teams will receive a behaviorally informed, bespoke training package and continuous intervention support during recruitment to the STAMINA trial (see Additional file 1) [24]. Training will last up to 3 h and will be delivered face-to-face or remotely depending on local policy and preference. The first training session will be delivered during site set-up and will be centred on four R’s: *R*ecognizing who is suitable for the STAMINA trial, *R*ecommending exercise in line with NICE guidance, *R*eferral to the STAMINA trial and *R*ecording research related information on the ADT log. Follow-up training will be delivered at 6–12 weeks following initiation of recruitment and will focus on supporting lifestyle behaviour change during routine clinic follow-up appointments. In parallel, ad hoc intervention support will be offered and regular feedback on referral performance will be provided via email. Full details of intervention development have been published [24].

#### The STAMINA lifestyle intervention (SLI)

SLI was developed in line with two overarching principles: (1) SLI will be offered as an intrinsic part of a man’s cancer care as opposed to an ‘add-on’ and (2) the intervention will be designed in a way to maximise both efficacy (likelihood of change) and effectiveness (accessibility and generalisability beyond urban areas).

SLI is a behaviourally informed, lifestyle intervention embedded into prostate cancer care to support delivery of NICE (NG131 1.4.19) guidance for people with prostate cancer on ADT (see Additional file 1). The SLI is focussed on supervised exercise training (primarily face-to-face) combining resistance and aerobic exercise with application of the behavioural skills learnt to other patient-negotiated lifestyle goals, including dietary modification. The SLI will be delivered in partnership with NH, a community-based, appropriately trained exercise provider, and will only be available to participants randomised to the intervention arm for 12 months.

SLI participants will be invited to an induction to exercise with a CES to explore their capability, opportunity, and motivation to exercise twice a week for 12 months. The supervised exercise sessions will include both aerobic (30–45 min) and resistance (up to 4 sets, 8–12 reps of major muscle groups) components as recommended by NICE (NG131 1.4.19 [19]) and in accordance with exercise programmes previously shown to be beneficial and safe to men on ADT (e.g. Bourke et al., 2014 [16]). The supervised exercise programme will be tailored to address individual requirements and will be delivered twice weekly for the first 12 weeks, initially one-to-one before transition to small groups (based on satisfactory progress). For the remaining 9 months of the programme, negotiated supervision will be offered (i.e. once a month maximum, once every 3 months minimum).

In parallel to supervised exercise sessions, behaviour change support will be delivered during programme reviews and catch-up calls between the CES and participant, and by completion of a behaviourally informed STAMINA diary. Programme reviews will be scheduled at 12 weeks, 6 months and 12 months following the induction session and will include a review of participant progress and a written summary report. Summary reports will be sent to the referring clinical team via email as part of the feedback loop and for discussion with the participant at their next routine clinic appointment. Catch-ups (face-to-face or over the phone) will also be organised with the CES and participant every 6 weeks to explore participant progress and address any barriers to intervention adherence (where applicable).

During the 12-month intervention period, SLI participants will be provided with a complimentary NH gym membership to support additional, independent exercise behaviour. SLI participants will also be offered one reduced price membership (~ 50%) for a family member to further support their exercise behaviour by addressing social support.

#### The exercise professional intervention

Community-based CESs and gym management/operational/front-of-house staff will receive a behaviourally informed, role-specific, bespoke training package with ongoing intervention support during delivery of the SLI (see Additional file 1). Level 1 training will comprise three online modules providing high-level information about prostate cancer, the SLI, patient confidentiality and operational procedures. All management, operational, sales, fitness and front-of-house staff will be invited to complete the online modules with a mandatory 80% pass mark. Staff responsible for the delivery of SLI (i.e. fitness managers, rehab specialists and personal trainers) will be invited to level 2 training which targets knowledge, confidence and skill related to delivering supervised exercise and behavioural support to men with prostate cancer. Level 2 training will be delivered face-to-face on site at NH over one full day. Ongoing tapered intervention support will be offered in the form of a weekly telephone call for up to 4 weeks followed by a monthly meeting with all CESs involved in the delivery of SLI. Full details of intervention development have been published [38].

### Criteria for discontinuing or modifying allocated interventions {11b}

The risks of the supervised exercise programme in men on ADT are minimal, with no increase in the risk of serious adverse events reported in a systematic review of RCTs evaluating the delivery of such programmes in these men [22]. All recruited men will continue to be under the care of their treating cancer clinician, who will be aware of their participation in the trial. In line with standard clinical care, cessation of the SLI at any time will be at the discretion of the clinicians or the participants themselves. Pauses to the programme will be accommodated for health-related reasons, holidays (up to 2 weeks maximum) and modification will be considered for individual cases (e.g. to remote sessions).

### Strategies to improve adherence to interventions {11c}

Intervention adherence will be monitored weekly by the research team at SHU by reviewing SLI participants’ attendance at supervised exercise sessions, to identify those who have dipped below 75% attendance. CESs at each NH site will upload data relating to attendance and completion of aerobic and resistance exercise components of the SLI after each session using a REDCap web-based reporting software. Researchers will download, compile, and clean the data on a weekly basis. The database will track adherence relative to the number of prescribed sessions to date and produce “alerts” once a SLI participant drops below 75% adherence. This alert with suggested actions will be sent onto the NH fitness manager who will identify and deliver behavioural support in line with the COM-B [30] model of behaviour change (i.e. data verification, identify required behavioural support and deliver behavioural support). If a participant’s attendance remains below 75%, a maximum two further alerts are sent. A record of all alerts sent and subsequent actions will be maintained.

### Relevant concomitant care permitted or prohibited during the trial {11d}

The protocol does not restrict participant access to usual care services. The usual care delivered at each site is documented at the point of site inclusion in the trial, at 12 months after the site opening and at 12 months after the last participant at the site is recruited. Participants also self-report their usual care received at 3-, 6- and 12-month post-randomisation. Should the trial team become aware of any new referrals to services, the nature of the referral will be established and discontinuation in the SLI considered.

### Provisions for post-trial care {30}

Should participants disclose anything to the trial team which puts them or anyone else at risk, the trial team may feel it necessary to report this to the appropriate persons. Additional exercise support outside of the SLI is not provided by CESs, and no specific aftercare is planned as part of the trial.

The NHS Sponsor is a member of the Clinical Negligence Scheme for Trusts (CNST) which provides indemnity cover for clinical negligent harm only. The Sponsor has an agreement in place with NH and therefore CNST indemnity extends to cover this service provider and their staff who have a duty of care to research participants.

### Outcomes {12}

Outcome data will be collected via participant self-report postal/online questionnaires and follow-up assessments with a local research nurse, or delegate, at 3-, 6- and 12-month post-randomisation. Supported completion of questionnaires by site staff can be requested by participants. If supported follow-up is required, wherever possible a research nurse, or delegate, who is blind to the participant’s allocation will offer telephone follow-up to participants who are unable to complete assessments independently. If site staff are subsequently unblinded, additional and subsequent data collection will be completed by an alternative researcher who is blinded to allocation.

#### Primary outcomes

Disease-specific QoL and fatigue at 12-month post-randomisation were measured by FACT-P [26] and FACIT-F [27]. The FACT-P [26] incorporates primary QOL domains: Physical Well-Being, Social/Family Well-Being, Emotional Well-Being, and Functional Well-Being with the addition of a prostate cancer-specific subscale. The FACIT-F [27] assesses perceptions of fatigue over the last 7 days.

#### Secondary outcomes

Secondary endpoints will be measured at 3-, 6- and 12-month post-randomisation and are as follows:

##### Questionnaire outcomes

- Physical, Social/Family, Emotional and Function wellbeing, assessed using FACT-P [26] subdomains
- Cancer-specific fatigue (FACIT-F [27].)
- Leisure time physical activity assessed using the Godin [28] questionnaire.
- Fear of recurrence and psychological distress markers assessed using FCR4 [29] and FCR7 [29].
- Adverse effects of ADT assessed using trial specific ADT Symptom Index.
- Perceptions of capabilities, opportunities and motivations to perform a target behaviour assessed by the COM-B [30] Questionnaire.

##### Physical measures

- Functional capacity and body composition assessed by blood pressure, chair sit-to-stand, waist and hip circumference and weight.

##### Safety

- Adverse events assessed by:
  - Number and proportion of RUSAEs
  - Number of RUSAEs per participant
  - Details of RUSAEs including severity
  - Number and proportion of deaths
  - Details of deaths including primary cause and timing

##### Health economics

- Generic quality of life assessed using EQ-5D-5L [31] questionnaire
- Costs of the intervention and of health care resource use
- Cost per incremental QALY using EQ-5D-5L [31] questionnaire
- Patient preferences and willingness to pay for exercise programmes as assessed by the DCE [32].

##### Moderator/mediators

Moderator and mediator variables which influence engagement with and benefit from the intervention will also be measured.

### Participant timeline {13}

**Fig. 1:**
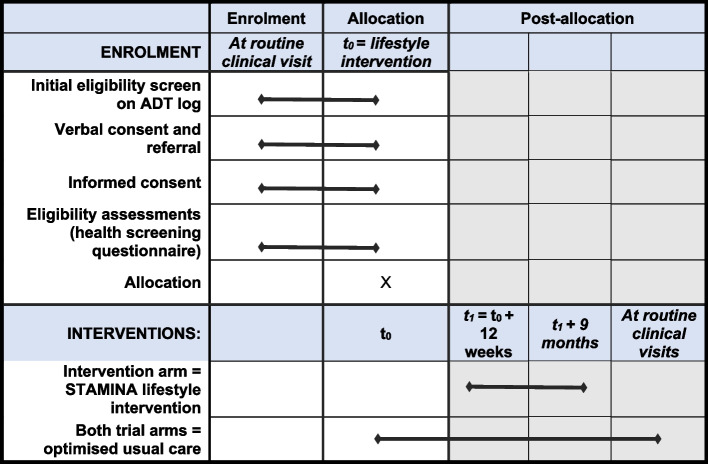
Schedule of enrolment and interventions

**Fig. 2:**
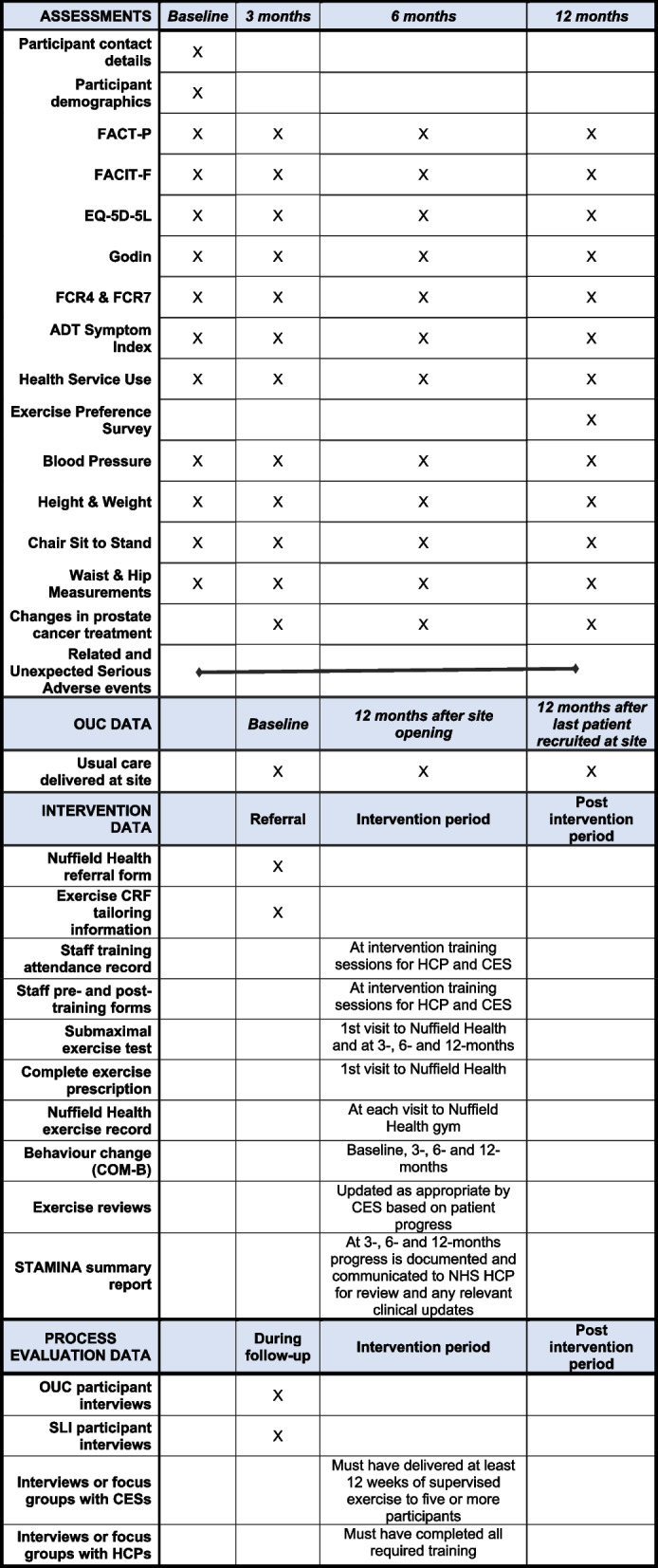
Schedule of assessments

The trial timeline for participants is presented in Fig. 1, outlining the schedule of enrolment and interventions, and in Fig. 2, outlining the schedule of assessments.

### Sample size {14}

A total sample size of 697 men (313 OUC, 384 SLI) is required to provide 90% power to detect a small to moderate effect size of either 0.33 in FACT-P [26] (8 point difference) or 0.35 in FACIT-F [27] (3 point difference) at the 5% significance level. Calculations assume a maximum standard deviation of 24 (FACT-P [26]) and 8.6 (FACIT-F [27]) and 30% loss to follow-up by 12 months [38]. Clustering at the level of the CES in the intervention arm is accounted for using an intracluster correlation coefficient (ICC) of 0.05 [39], a coefficient of variation of 0.6 to account for variation in the number of men each CES will deliver the intervention to, an average cluster size of 6 participants (range 1 to 14) per CES and 64 CES.

The sample size calculation takes into account the correlation between the two primary endpoints based on methodology by Micheaux et al. [40]. A correlation between FACT-P [26] and FACIT-F [27] of 0.7 (95% confidence internal (CI): 0.58–0.78) was observed at baseline in the pilot data [16]. The lower bound of the CI for the correlation estimate (0.58) was incorporated into the calculations. After accounting for multiple testing with correlated continuous multiple primary endpoints, the adjusted alpha level is 0.02768.

### Recruitment {15}

To ensure recruitment of 697 men within an 18-month period, the internal pilot green progression criteria for recruitment was set as at least 4 men per site per month from a minimum of 12 NHS Trusts based on a minimum of 40 men from each site, per year, over a total recruitment period of 18 months. Additional sites will be added during the recruitment period to deliver the overall sample size of 697 if required.

## Assignment of interventions: allocation

### Sequence generation {16a}

Participants will be randomised on a 5:4 basis to receive either SLI or OUC. A computer-generated minimisation programme incorporating a random element will be used to ensure arms are well-balanced for the following stratification factors:

- Age: < 70 years OR ≥ 70 years
- Duration on ADT: ≤ 12 weeks OR > 12 weeks
- Receiving chemotherapy and/or novel androgen receptor inhibitors: yes OR no
- Receiving radiotherapy: yes OR no

### Concealment mechanism {16b}

Randomisation will be performed using the Leeds CTRU automated 24-h randomisation service, which can be accessed via the web and ensures allocation concealment. Personal usernames and passwords required to access the randomisation system will be provided to sites when all relevant trial approvals are in place.

### Implementation {16c}

Following participant consent, confirmation of eligibility and collection of baseline data, a member of the SHU research team will randomise the participant. The principal investigator and relevant local research team at the site will receive an automated email confirmation of successful randomisation, highlighting subsequent tasks required and omitting randomisation allocation details. Participants will be contacted via letter/email to confirm their randomisation allocation. The research team at SHU will refer participants randomised to SLI to NH to initiate the exercise programme.

## Assignment of interventions: blinding

### Who will be blinded {17a}

Participants and NH staff will be aware of treatment allocation, but researchers involved in data collection, general practitioners (GP) and clinical teams will be blind to the allocation. Analysis will be conducted unblind to allocation according to pre-specified analysis plans.

It is not possible to blind participants or those involved in the delivery of the intervention in studies evaluating exercise/lifestyle interventions.

CTRU will regularly monitor for bias by reviewing participant characteristics (e.g. stratification factors, number of comorbidities and other characteristics as deemed appropriate) by the treatment arm to check for imbalances. CTRU and the trial management group (TMG) will monitor instances of unblinding to review for systematic errors that could impact upon trial integrity and elevate concerns to the programme steering committee (PSC).

### Procedure for unblinding if needed {17b}

Trial participation will be recorded in participants’ hospital notes and with their GP. This will not reveal allocation but will detail where further information can be gained if required. A central list of participants’ randomisation allocations will be maintained at SHU and at CTRU. We do not foresee a situation where allocation needs to be revealed outside of regular office hours.

## Data collection and management

### Plans for assessment and collection of outcomes {18a}

The schedule for participant assessments is summarised in Fig. 2: Schedule of assessments.

Baseline data for consenting patients will be collected by the research team at SHU from the participant directly. The local researcher at the participating site will collect data from medical records (e.g. blood pressure). Participants will self-complete all remaining assessments. Where inclusion in the trial is deferred until after completion of a course of chemotherapy, baseline assessments will be repeated before randomisation.

At 3-, 6- and 12-month post-randomisation, follow-up questionnaires will be completed by participants online or by post. Supported follow-up by site staff can be requested by participants. If questionnaires are not returned on time, up to three reminders may be sent via post, email, or text depending on the participants’ preferred method of contact. Where relevant, local researchers at participating sites will be asked by CTRU to confirm survival status and current address.

At 3-, 6- and 12-month post-randomisation, local researchers at participating sites will collect data from participants directly and from their medical records (e.g. blood pressure, changes in prostate cancer treatment). Any RUSAEs will also be collected.

Local researchers at participating sites will receive training on the completion of all trial-specific assessments as part of trial initiation, to ensure standardised completion. CTRU will chase missing and discrepant data as appropriate. Data collection forms are available on request at ctru-dataaccess@leeds.ac.uk.

### Plans to promote participant retention and complete follow-up {18b}

Initial/reminder letters, texts and emails will be used to maximise questionnaire data return at all timepoints. Participants may request support in the completion of questionnaires by site staff or the trial team in advance or during routine telephone reminders. The TMG will monitor questionnaire return rates overall and by the treatment arm.

A postcard from the PPI group will be sent to participants at 11 months to remind them their 12-month questionnaire will arrive soon and to encourage engagement with the project. A poster from the PPI group will also be sent to participants alongside their 12-month questionnaire to provide thanks, encouragement to complete the final questionnaire, and to remind participants how to receive the study results.

Participants will remain free to withdraw at any time from the trial without giving reasons and without prejudicing their further treatment. If a participant withdraws consent to participate, clarification will be sought on whether withdrawal is because of participation in the intervention, questionnaire completion or ongoing access to health records and data processing. Data collected up to the point of withdrawal will be included in the final trial analysis. This will be made clear to the participants at the time of consent and when they withdraw from the trial.

### Data management {19}

All data collection forms transferred to or from the CTRU will be coded with the participant’s trial number, initials and site code. Data will be held securely on paper and electronically at CTRU, Sheffield Hallam University and at NH. All relevant Standard Operating Procedures, Guidelines and Work Instructions in relation to data management, processing and analysis of data will be followed. CTRU will provide sites with an electronic file to safely maintain essential trial documentation. Interviews for the process evaluation will be audio recorded and professionally transcribed, with any identifiable information removed. Audio files will be securely transferred in encrypted format and securely stored at Queen Mary University London, Sheffield Hallam University and Bristol.

### Confidentiality {27}

All information collected during the trial will be kept strictly confidential, complying with all aspects of the 2018 Data Protection Act [41]. Appropriate storage, restricted access and disposal arrangements of personal and clinical details of participants will be put in place. At the end of the trial, sites will archive all trial data until written permission for confidential destruction is provided by the Sponsor. The Trial Master File and documents held by the CTRU will be archived at a secure facility at the University of Leeds.

### Plans for collection, laboratory evaluation and storage of biological specimens for genetic or molecular analysis in this trial/future use {33}

Not applicable; no samples were collected.

## Statistical methods

### Statistical methods for primary and secondary outcomes {20a}

A detailed Statistical Analysis Plan will be written before any analyses are undertaken. Final analysis will be conducted once all available outcome data is received. All analyses will be conducted on the intention-to-treat (ITT) population, with all participants included in the analysis according to their randomisation allocation, and regardless of non-adherence to the intervention or withdrawal from the trial. Blinded interim reports will be presented to the PSC containing descriptive summaries of recruitment, follow-up, safety and data quality.

#### Primary endpoint analysis

Primary analysis will compare mean FACT-P [26] and FACIT-F [27] scores at 12-month post-randomisation between the trial arms using partially nested mixed-effects linear regression models to account for clustering of outcomes in the intervention arm due to the nesting of participants within CESs [42]. The models will be adjusted for the stratification factors and other participant-level covariates expected a priori to be associated with the outcome of interest. Each endpoint will be tested against the calculated adjusted alpha level 0.02768. If a statistically significant result is observed in either FACT-P [26] or FACIT-F [27], the SLI will be deemed clinically different to OUC. Results will be expressed as estimated mean differences with 97.232% confidence intervals, *p*-values and ICCs. Model diagnostics will be visually assessed to check the underlying assumptions of the model and alternative methodology will be used if required.

#### Secondary endpoint analysis

Summary statistics will be presented for each time point by arm for secondary outcomes FACT-P [26] (overall and domain-specific), FACIT-F [27], leisure time physical activity (Godin [28]), fear of recurrence (FCR4 [29] and FCR7 [29]), adverse effects of ADT (ADT Symptom Index), functional capacity and body composition (blood pressure, chair sit-to-stand, waist and hip circumference and weight). Means, standard deviations, medians, minimum, maximum, quartiles and ranges will be presented for continuous variables and frequencies and percentages for categorical variables. Secondary endpoints will be analysed using the same approach as for the primary outcome with the relevant model for the type of outcome variable. Safety endpoints will be analysed descriptively between arms and no formal statistical comparisons will be made.

Moderators and mediators will be identified. Whether the treatment effect differs depending on the pre-specified baseline characteristics of the participant will be explored.

#### Internal pilot

Descriptive analysis of the internal pilot against progression criteria (Table 1) together with an examination of the sample size assumptions will take place after 12 months of recruitment.

**Table 1:**
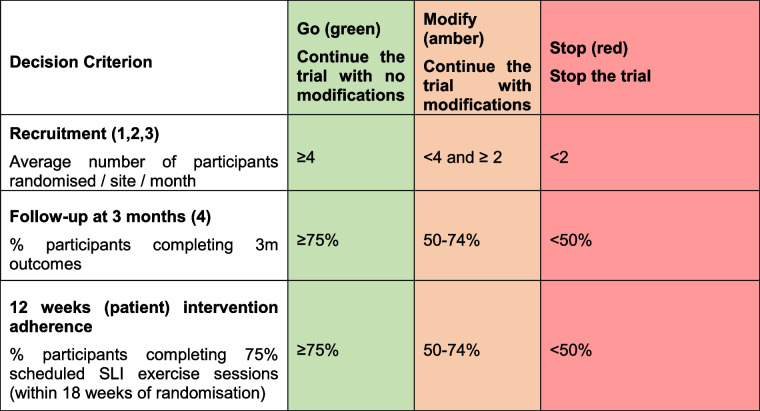
Progression criteria

^1^Data from August 2022 onwards will be used for analysis of this criterion to allow for a sufficient number of sites to be open and to ensure the trial has reached a steady rate of recruitment

^2^This target is set at a gym level. Therefore, for sites with 2 gyms, the criteria are green ≥ 8, amber < 8 and ≥ 4, and red < 4

^3^Based on 12 recruiting sites

^4^FACT-P [26] and FACIT-F [27] only

### Interim analyses {21b}

No formal interim analyses of outcomes will be undertaken.

### Methods for additional analyses (e.g. subgroup analyses) {20b}

No sub-group analyses are planned.

### Methods in analysis to handle protocol non-adherence and any statistical methods to handle missing data {20c}

We will explore missing data patterns and reasons for missingness to guide the assumptions around missing data. If it can be assumed that data is missing at random (MAR), the primary ITT analysis will use multiple imputation, enabling inclusion of all randomised participants. If data cannot be assumed MAR we will explore other more complex methods for the primary analysis such as pattern mixture modelling.

### Plans to give access to the full protocol, participant-level data and statistical code {31c}

Anonymised data supporting this work are available on reasonable request. All requests will be reviewed by relevant stakeholders, based on the principles of a controlled access approach. Requests to access data should be made to CTRU-DataAccess@leeds.ac.uk in the first instance.

## Economic evaluation

The economic evaluation will use data at 12 months and will adopt the NICE reference case employing a primary endpoint of cost per incremental QALY [43]. The primary perspective will be the health and personal social service provider, but will include supplementary analyses incorporating patient/caregiver costs (e.g. out-of-pocket expenses in attending exercise sessions).

Costs will include the costs of the interventions, costs of any additional exercise equipment and exercise facility access purchased or provided and any additional healthcare resources used (including primary and secondary care). Unit costs will be taken from the Personal Social Services Research Unit report and NHS Reference costs. Health outcomes will be assessed by the EQ-5D-5L [31] at follow-up but we will also explore basing utility on the FACT-P [26] and/or FACIT-F [27] measures (via mapping) depending on the availability of suitable mapping algorithms or direct valuation tariffs.

A Health economic analysis plan (HEAP) will be developed prior to analysis. The HEAP will be reviewed by the project team and an external health economist. The primary analysis will adopt an ITT approach but with additional pre-specified analyses conducted to evaluate the impact of intervention compliance on cost-effectiveness.

We will generate ICERs per QALY at 3, 6 and 12 months. We will use seemingly unrelated regression to account for the correlation between costs and QALYs and within this use relevant covariates to adjust for any imbalance across arms. We will use robust standard errors to account for any potential clustering in outcomes by CES in the intervention arm. The analytical process will also incorporate the strategy for handling missing data (e.g. multiple imputation, if appropriate) and the variance–covariance matrix used to generate 10,000 ICERs. The latter simulations will be plotted on the cost-effectiveness plane and help generate the cost-effectiveness acceptability curve to characterise the level of sampling uncertainty in the analysis.

A decision-analytic model will be developed to extrapolate costs and benefits over a lifetime horizon following consultation with clinical experts and patients. It will likely be a Markov Model. We will use trial data and targeted literature reviews to derive the model parameters and explore using resources such as the National Prostate Cancer Audit. We will generate lifetime ICERs for SLI vs. OUC. We will assume a cost-effectiveness threshold of £20,000 per QALY gained. We will generate net monetary benefit (NMB) and conduct extensive one-way and scenario deterministic sensitivity analyses. We will explore, the impact of different intervention costs, engagement and effectiveness decay over time on the estimates of cost-effectiveness. A probabilistic sensitivity analysis will capture the total parameter uncertainty in the model. Results from this will be presented in the form of cost-effectiveness planes, NMB distributions and cost-effectiveness acceptability frontiers [44].

We will use information from the discrete choice experiment on men’s willingness to pay and likely uptake for extended (and alternative) programmes to explore alternative funding strategies. These will be incorporated in supplementary analysis exploring the cost-effectiveness of alternative programmes.

## Process evaluation

The mixed methods process evaluation will be undertaken based on the Medical Research Council (MRC) guidance [45] for process evaluations and informed by the framework of Linnan and Steckler [46]. Quantitative and qualitative data will be collected to address five objectives. Data will be collected throughout the whole trial period to explore different stages of the intervention/trial. Further detail will be presented in a separate process evaluation protocol manuscript (*in preparation*).

## Oversight and monitoring

### Composition of the coordinating centre and trial steering committee {5d}

The coordinating centre (SHU) is responsible for intervention training and undertaking individual participant research activities, including consent, eligibility and data collection. The CTRU at the University of Leeds is responsible for set up, implementation and monitoring of trial conduct; data management and statistical design, analysis and reporting and is where the trial and data management and statistics teams are based.

The PSC will provide overall supervision of the trial and are responsible for monitoring trial progress and providing public, clinical, and professional advice. It will include an Independent Chair, at least two other independent members, and a PPI representative. The Committee will meet annually as a minimum.

The programme management group (PMG) oversees the STAMINA Programme Grant and includes the chief investigator, co-applicants and co-investigators. The PMG will oversee the whole programme of studies and meet twice annually as a minimum.

The TMG includes the chief investigator, trial and process evaluation leads, key co-applicants, the CTRU delivery team, the SHU research team, a PPI representative and other key external members of staff involved in the trial. The TMG will oversee trial set-up; on-going management; promotion of the trial; and the interpretation and publishing of the results. The TMG will meet quarterly as a minimum.

### Composition of the data monitoring committee, its role and reporting structure {21a}

For a trial of this nature, a separate Data Monitoring and Ethics Committee is not required. Rather, the PSC will adopt a safety function, with the constitution of a sub-committee to review safety issues, where necessary.

Summary safety data will be provided to the PSC to determine patterns and trends of events or to identify safety issues, which would not be apparent on an individual case basis.

### Adverse event reporting and harms {22}

Adverse events fulfilling the definition of related and unexpected are reportable in this trial (resulting from the administration of any of the research procedures). All related and unexpected serious adverse events will be reviewed by the principal investigator and reported to CTRU within 24 h of becoming aware. They will be subject to expedited reporting to the Sponsor and the Research Ethics Committee (REC) within 15 days. Where the principal investigator is unable to decide whether or not the serious adverse event is either ‘related’ or ‘unexpected’, the event should be reported to the chief investigator, who will liaise with the coordinating centre and CTRU in arriving at a decision. Events will be followed up until the event has been resolved or a final outcome has been reached.

### Frequency and plans for auditing trial conduct {23}

Investigators are required to promptly notify the CTRU if there is a breach of the protocol or of the conditions or principles of Good Clinical Practice which is likely to affect to a significant degree the safety or physical or mental integrity of the trial subjects, or the scientific value of the research.

The CTRU/Sponsor (or delegate) or regulatory authority reserves the right to conduct intermittent source data verification on a sample of participants. Source data verification will involve direct access to patient notes at the participating hospitals, and other relevant investigation reports.

### Plans for communicating important protocol amendments to relevant parties (e.g. trial participants, ethical committees) {25}

Protocol amendments will be processed in line with REC and Health Research Authority (HRA) guidelines. Investigators will be notified and asked to confirm ongoing capacity and capability.

### Dissemination plans {31a}

Trial results will be disseminated nationally and internationally to extend reach to participants, HCPs, CESs, academic researchers, the public and other key stakeholders on completion of the trial. Dissemination activities include participation in conferences, publication in peer-reviewed journals, delivery of webinars and organisation of PPI events. To maintain the scientific integrity of the trial, data will not be released prior to the end of the trial, either for publication or oral presentation purposes, without the permission of the PSC or the chief investigator and trial leads.

## Discussion

STAMINA is a multi-centre, two-arm RCT examining the clinical and cost-effectiveness of the SLI compared to OUC at a 12-month follow-up. In addition, a mixed methods process evaluation and health economic evaluation will be conducted.

Men on ADT for prostate cancer experience persistent adverse effects from treatment that are often debilitating and can be experienced lifelong [2]. To date, supervised exercise is the only evidence-based intervention to address the harms caused by treatment, but previous research has failed to demonstrate sustained clinical benefit and behaviour change maintenance [22]. Moreover, NHS provision of supervised exercise, as recommended nationally and internationally, is almost non-existent due to behavioural and implementation barriers [21]. Therefore, a major opportunity exists to address this unmet need and to improve the QoL and reduce fatigue of men on ADT for prostate cancer through STAMINA.

The SLI is a behaviourally informed complex intervention designed with methodological rigour. The intervention elements are underpinned by behaviour change theory, implementation science and evidence to optimise long-term behaviour change [47]. Intervention development took an iterative approach with continuous feedback from stakeholders and PPI to enhance acceptability and implementation of the proposed service level change to clinical care delivery [48]. The complex intervention addresses professional (i.e. HCPs and CESs) and patient behaviour change and will be the first of its kind to be delivered as part of routine care to bridge the gap between research, policy and practice and maximise future scale-up. Moreover, STAMINA benefits from the addition of a novel ‘safety to exercise’ check for all participants to maximise safety and a unique approach to patient recruitment. Men with prostate cancer will be identified in routine NHS clinics but recruited by non-NHS researchers to reduce burden on NHS staff during unprecedented times (i.e. the COVID-19 pandemic).

The trial has the potential to significantly impact a large cohort of men with prostate cancer on ADT due to its rigorous approach to intervention development and the trial design, e.g. the broad eligibility criteria, long-term follow-up and because the prevalence and survival time for prostate cancer is continuing to rise [1]. Eight out of ten men now survive the disease for 10 years or more [1] and thus the patient and professional demand for such intervention continues to grow. The process evaluation will be essential for understanding if, why and how the intervention works in the real-world context and will contribute towards recommendations for future clinical care [49]. Moreover, if found to be effective, STAMINA could produce a blueprint for the integration of supervised exercise and behavioural support into other cancer and/or clinical services.

To date, the SLI has demonstrated good feasibility, fidelity, safety and acceptability when embedded into routine NHS care as part of a feasibility study [25]. This definitive pragmatic trial will evaluate the long-term clinical and cost-effectiveness of SLI when delivered in the real world and when compared to OUC.

## Trial status

Protocol v10.0 05/07/2023. Recruitment began in January 2022 and will be ongoing until all interviews for the process evaluation are complete (~ January 2024) .

### Supplementary Information

**Additional file 1.** This file contains three tables to describe each component of the intervention using the TIDieR framework: 1) STAMINA Lifestyle Intervention., 2) Healthcare Professional Intervention. and 3) Exercise Professional Intervention.

## Data Availability

The Sponsor (Sheffield Teaching Hospitals NHS Foundation Trust), Coordinating Centre (Sheffield Hallam University) and the Clinical Trials Unit (CTRU, University of Leeds) will act as Data Controllers. Requests to access anonymised data should be made to CTRU-DataAccess@leeds.ac.uk in the first instance.

## Abbreviations

ADT: Androgen deprivation therapy
CES: Clinical Exercise Specialist
CI: Confidence interval
CNST: Clinical Negligence Scheme for Trusts
COM-B: Capability, Opportunity, Motivation-Behaviour
CRUK: Cancer Research UK
CTRU: Leeds Clinical Trials Research Unit
EQ-5D-5L: EuroQol 5 Domains 5 Levels
DCE: Discrete choice experiment
FACIT-F: Functional Assessment of Chronic Illness Therapy – Fatigue
FACT-P: Functional Assessment of Cancer Therapy – Prostate
FCR: Fear of Cancer Recurrence (questionnaire)
GP: General practitioner
HCP: Healthcare professional
HEAP: Health Economic Analysis Plan
HRA: Health Research Authority
ICC: Intracluster correlation coefficient
ICER: Incremental cost-effectiveness ratio
ITT: Intention-to-treat
NIHR: National Institute for Health Research
NH: Nuffield Health
NHS: National Health Service
NICE: National Institute of Health and Care Excellence
NMB: Net monetary benefit
OUC: Optimised usual care
MAR: Missing at random
MRC: Medical Research Council
PCUK: Prostate Cancer UK
PMG: Programme management group
PPI: Patient and Public Involvement
PSC: Programme Steering Committee
QALY: Quality-adjusted life year
QoL: Quality of life
RCT: Randomised controlled trial
REC: Research Ethics Committee
RUSAE: Related and unexpected serious adverse event
SHU: Sheffield Hallam University
SLI: STAMINA lifestyle intervention
TMG: Trial management group

## Footnotes

1 Where a man is due to start Taxane based chemotherapy in conjunction with ADT, an initial health questionnaire will be conducted but inclusion in the trial will be deferred until after completion of the course of chemotherapy (typically 18–20 weeks). Baseline assessments will be repeated before randomisation where necessary.
