# Supplementary material for: Supported exercise TrAining for Men wIth prostate caNcer on Androgen deprivation therapy (STAMINA): study protocol for a randomised controlled trial of the clinical and cost-effectiveness of the STAMINA lifestyle intervention compared with optimised usual care, including internal pilot and parallel process evaluation
Source: Trials. 2024 Apr 12;25:257. doi: 10.1186/s13063-024-07989-y (PMC11010375; doi:10.1186/s13063-024-07989-y)
Supplement: Supplementary file 1 — Additional file 1. This file contains three tables to describe each component of the intervention using the TIDieR framework: 1) STAMINA Lifestyle Intervention., 2) Healthcare Professional Intervention. and 3) Exercise Professional Intervention. [file 13063_2024_7989_MOESM1_ESM.docx]

**Table 1: TIDieR Framework of the STAMINA Lifestyle Intervention**

| **Name** | STAMINA - Supported exercise TrAining for Men with prostate caNcer on Androgen deprivation therapy |
| --- | --- |
| **Why** | The National Institute for Health and Care Excellence (NICE) recommend that people on androgen deprivation therapy (ADT) for prostate cancer should be offered twice weekly aerobic and resistance exercise for 12 weeks to improve cancer specific fatigue and quality of life. However, these guidelines are not being delivered in the NHS highlighting the ongoing challenge of translating evidence into complex healthcare systems. |
| **What** | The STAMINA Lifestyle Intervention will include:   1. a recommendation to exercise from a healthcare professional from Urology or Oncology. 2. a safety to exercise check 3. a referral to Nuffield Health. 4. an information pack and behaviourally informed diary to track progress. 5. an induction to the gym. 6. a tailored exercise programme. 7. twice a week supervised aerobic and resistance exercise for 12 weeks. 8. tapered supervised exercise between month 4 and 12. 9. quarterly progress reviews and behavioural support. |
| **Who provides** | The STAMINA Lifestyle Intervention will be offered to eligible participants as part of a pragmatic randomised controlled trial (RCT). Participants will be identified and recruited from up to one of twenty NHS trusts involved in delivering the RCT. Participants randomised to receive the intervention will be referred to Nuffield Health. Nuffield Health is the UK’s largest healthcare charity with a network of 37 hospitals, 114 fitness and wellbeing centres and workplace wellbeing facilities. |
| **How** | The STAMINA Lifestyle Intervention is designed to be delivered face-to-face one-to-one and face-to-face to small groups (maximum of 5 people). |
| **Where** | The STAMINA Lifestyle Intervention has been designed to be delivered at up to 20 Nuffield Health fitness and wellbeing centres as part of the RCT. More specifically, on the gym floor and in clinic/ consultation rooms. |
| **When and how much** | STAMINA Lifestyle Intervention participants will receive a 12-month exercise programme as part of the pragmatic RCT. In line with recommendations from NICE, participants will be offered supervised exercise twice weekly for 12 weeks based on the patient and clinical exercise specialist’s availability. Supervision will be reduced for the remaining 9 months of the programme, but participants will be encouraged to continue exercising twice per week independently.  The full STAMINA exercise prescription includes:   - Frequency – twice per week - Intensity – moderate to hard - Type – aerobic and resistance - Time – 30-45 minutes aerobic and up to 4 sets, 8 – 12 reps resistance exercise.   In parallel, participants will be provided with a behaviourally informed STAMINA diary (for self-monitoring) and progress reviews including behavioural support underpinned by the Theoretical Domains Framework.   - Week 4 review – feedback and rewards - Week 6 review – feedback and goal setting - Week 12 review – feedback, social support, and habit formation - Week 26 review – feedback and monitoring - Week 39 review – feedback and goal setting - Week 52 review – feedback and action planning |
| **Tailoring** | The exercise programme will be tailored based on each participants capability, opportunity, and motivation to exercise. |
| **Modifications** | The STAMINA Lifestyle Intervention can be modified to be delivered remotely using Microsoft Teams for participants who have difficulty attending the gym due to treatment side effects or in extreme cases. |
| **How well** | Intervention acceptability will be assessed in a parallel process evaluation via semi-structured interviews. |

**Table 2: TIDieR Framework of the Healthcare Professional Intervention**

| **Name** | STAMINA Healthcare professional training - Supported exercise TrAining for Men with prostate caNcer on Androgen deprivation therapy |
| --- | --- |
| **Why** | The National Institute for Health and Care Excellence (NICE) recommend that people on androgen deprivation therapy (ADT) for prostate cancer should be offered twice weekly aerobic and resistance exercise for 12 weeks to improve cancer specific fatigue and quality of life. However, these guidelines are not being delivered in the NHS highlighting the ongoing challenge of translating evidence into complex healthcare systems. Maintaining exercise behaviour is difficult. The healthcare professional training aims to address this throughout the patient journey partly via the behaviour of the HCP. |
| **What** | Healthcare professionals involved in the prostate cancer care pathway will be provided with a behaviourally informed training package which will enable them to;   - embed NICE recommendations into NHS prostate cancer care; - endorse, recommend, and support lifestyle changes for men on ADT during routine clinical contact; - elicit beliefs, provide support, and outline patient concerns.   The aims are to encourage uptake of and adherence to the intervention.  The training will include a PowerPoint presentation, handouts of the training slides, worksheets and prompts for use in clinics. Facilitators will use a training manual based on the PowerPoint slides.  The training (underpinned by the Theoretical Domains Framework) will provide:   - An introduction to NICE NG131 1.4.19 recommendations (*knowledge*). - An overview of the benefits of exercise for men with prostate cancer on Androgen Deprivation therapy (knowledge, *beliefs about consequences*) - Identify clinical roles within the team to aid the implementation of the NICE recommendations (*professional role*). - Provide HCPs with the appropriate skills in terms of behaviour change techniques to use to support this patient group with exercise (*skill*). - Provide HCPs with the information on how to make referrals for exercise, what information to hand out to patients and how secure communication will take place with NH and the NHS (*environmental context*). |
| **Who provides** | The healthcare professional training will be facilitated by two members of staff, including a health psychologist, a behavioural science research fellow, a psychology research fellow, and a research nurse. |
| **How** | The healthcare professional training is designed to be delivered face-to-face. The follow up training is designed to be delivered remotely. |
| **Where** | NHS Hospital sites or remotely using Microsoft teams. |
| **When and how much** | The healthcare professional training will be delivered during the site set up of the randomised controlled trial, the training duration is 2-3 hours. Follow up training will be delivered approximately 8 weeks after the NHS recruitment start date, this will be 1 hour in duration. |
| **Tailoring** | NA |
| **Modifications** | If required by clinical teams the healthcare professional training can be delivered remotely using Microsoft teams. |
| **How well** | Treatment fidelity will be assessed in parallel process evaluation using a Theoretical Domains Framework questionnaire. Intervention acceptability and implementation will be explored in semi-structured interviews and field notes. |

**Table 3: TIDieR Framework of the Exercise Professional Intervention**

| **Name** | Community-based exercise professional training package: Supporting exercise behaviour of men on androgen deprivation therapy (ADT) for prostate cancer |
| --- | --- |
| **Why** | The National Institute for Health Excellence (NICE) recommend that people on ADT are offered twice weekly aerobic and resistance exercise to improve cancer specific fatigue and quality of life. However, these guidelines are not being met. Community-based exercise professionals are well suited to deliver these recommendations however due to their limited training and experience of working with clinical populations, further training is required. |
| **What** | Community-based exercise professionals will be provided with a training manual, PowerPoint slides, video examples of all behaviours, case studies and associated worksheets to facilitate activities centred on tailoring exercise, delivering supervised exercise, data recording, progress reviews and behavioural support.  **Level 1 training**  Module 1: Introduction to prostate cancer and exercise  This module provides introductory content on the symptoms, prevalence, treatments and side effects of prostate cancer and the evidence-base of exercise as a treatment component.  Module 2: Introduction to the STAMINA Lifestyle Intervention  This module provides an overview of the STAMINA Lifestyle Intervention, including its core components and types, frequency, duration, and mode of each session to be delivered during the 12-month intervention.  Module 3: Operationalising the STAMINA programme  This module provides content on scheduling patient appointments and the referral/ communication pathway between the exercise professionals and NHS clinical team.  **Level 2 training**  Module 1: Overview of online content  This module focusses on developing an understanding of the target population (i.e., men with PCa on ADT), the core components of SLI and the role of the exercise professional.  Module 2: Contacting the patient  This module provides step-by-step instruction and demonstration of the first patient contact. Discussion is centred on beliefs about patient capability, opportunity and motivation, strategies to address possible barriers and what makes a good consultation.  Module 3: Delivering the STAMINA induction  This module covers the skills and knowledge required to deliver the STAMINA induction including delivery of a sub-maximal exercise test and tailoring the exercise prescription for clinical populations. Evidence based information is provided as well as skill-based learning with feedback (e.g., instruction, demonstration, behavioural practise).  Module 4: Delivering the exercise prescription  This module is centred on delivering and recording tailored exercise one-to-one and in small groups to a maximum of 5 people. This module incorporates discussion, case study examples and skill-based learning (including feedback and graded tasks).  Module 5: Reviewing patient progress  This module prepares exercise professionals to review patient progress to determine suitability to transition from one-to-one to group supervision (at week 2, 4, 6), to negotiate tapered supervision and repeat the fitness check (at week 12, 26 and 52).  Module 6: Supporting behaviour change  This module focusses on behaviour change theory and behaviour change techniques to support initiation and maintenance of exercise, as well as skills to address ambivalence and resistance to exercise. Case study examples and skill-based training is included. |
| **Who provides** | Community-based exercise professionals will receive training facilitated by two members of staff. The lead facilitator is a Behavioural Science Senior Research Fellow with a background in Sport and Exercise Science, Psychology and Public Health. The co facilitator, from Nuffield Health, has a background in personal training, exercise physiology and experience designing and delivering rehabilitation programmes for clinical populations. |
| **How** | The training package is designed to be delivered in two sections: online (Level 1) and face-to-face (Level 2). Level 1 is to be completed individually whereas Level 2 training is delivered face-to-face in small groups, ranging from 2 -15 people. |
| **Where** | The community-based exercise professional intervention is designed to be delivered on site at community exercise gyms with access to an education room (tables, chairs, projector and screen) and equipment on the gym floor for practical sessions. |
| **When and how much** | The community-based exercise professional intervention is designed to be completed once by each individual. Instructions for level 1 training are provided approximately four weeks before the scheduled level 2 training to provide sufficient time for completion. Staff must pass level 1 training with a minimum of 80% before attending level 2 training. |
| **Tailoring** | NA |
| **Modifications** | The module order may have to be amended slightly based on training location and access to the gym floor though all content will always be covered. |
| **How well** | Fidelity, acceptability, and implementation will be explored in a parallel process evaluation via observations, semi-structured interviews, audio recorded consultations and field notes. |
